# Physiological underarousal as a mechanism of aggressive behavior in university athletes with a history of concussion

**DOI:** 10.1002/brb3.1038

**Published:** 2018-07-20

**Authors:** Caitlyn Gallant, Nicole Barry, Dawn Good

**Affiliations:** ^1^ Department of Psychology Brock University St. Catharines ON Canada; ^2^ Centre for Neuroscience Brock University St. Catharines ON Canada

**Keywords:** aggression, arousal, athletes, concussion history

## Abstract

**Introduction:**

Research has indicated that athletes who engage in high‐risk athletic activities, such as football and hockey, have riskier personalities than their low‐risk and nonathlete counterparts (Ahmadi et al., 2011, Procedia Soc Behav Sci, 30 and 247–251; Zuckerman, 1983, Biological bases of sensation seeking, impulsivity, and anxiety, Lawrence Erlbaum Assoc Inc.). For instance, increased sensation‐seeking and aggression are common in high‐risk athletes, rendering these individuals more likely to sustain a subsequent injury, such as concussion. Elevated levels of certain personality traits, including impulsivity and aggression, have also been observed after concussion (Goswami et al., 2016, Brain Struct Funct, 221 and 1911–1925). The purpose of this study therefore was to determine whether aggressive behavior in university athletes may be accounted for, in part, by a history of concussion, rather than exclusively athletic status.

**Methods:**

Using a quasi‐experimental design, 66 university students (*n *=* *18 nonathletes, *n *=* *24 low‐risk athletes, *n *=* *24 high‐risk athletes) with (*n *=* *27) and without a history of concussion (*n *=* *39) completed the Buss & Perry Aggression Questionnaire (BPAQ; Buss & Perry, 1992, J Pers Soc Psychol, 63 and 452) and provided electrodermal activation (EDA) as an index of physiological arousal.

**Results:**

It was found that decreased physiological arousal among students with a history of concussion was associated with greater endorsement of physical aggression. Moreover, athletic status did not account for this pattern of aggression, as athletes and nonathletes did not differ in terms of self‐reported aggressive tendencies.

**Conclusions:**

Physiological compromise after concussive injury may act as an independent mechanism of aggressive behavior in athletes beyond factors, such as athletic status.

## 1 INTRODUCTION

It is well established that athletes engage in higher levels of risk‐taking compared to nonathletes (e.g., Hingson, Heeren, Zakocs, Kopstein, & Wechsler, 2002; O'Malley & Johnston, 2002); this risk‐taking behavior often includes acts of aggression (Lenzi et al., 1997; O'Brien et al., 2012; Tenenbaum, Singer, Stewart, & Duda, 1997), as well as violent behavior (e.g., Koss & Gaines, 1993). Indeed, in a meta‐analysis by Sønderlund et al. (2014), athletes exhibited significantly higher levels of violence and aggression compared to nonathletes across 10 of the 11 studies reviewed. Similarly, it has been demonstrated that athletes endorse higher levels of disinhibition, irritability, and aggressiveness than their nonathlete counterparts (Bara Filho, Ribeiro, & García, 2005), even in nonsporting contexts (Bredemeier, Shields, Weiss, & Cooper, 1986).

Other investigations have reported differences in aggressive behavior among contact and noncontact athletes (e.g., Ziaee, Lotfian, Amini, Mansournia, & Memari, 2012), suggesting that the nature of contact sports engenders aggression. Although findings from this literature have been inconsistent (Keeler, 2007; see Kimble, Russo, Bergman, & Galindo, 2010 for a review), proponents of this perspective assert that aggressive behavior is reinforced and maintained in contact/high‐risk sports when athletes learn that aggression can have instrumental value (Butt, 1987; Weiner, 1974). In turn, these learned behaviors are generalized to nonsporting contexts and used to obtain external rewards. To illustrate this idea, Huang, Cherek, and Lane (1999) used the Point Subtraction Aggression Paradigm (PSAP; Cherek, 1981) and compared male contact, and noncontact, athletes. Interestingly, it was found that contact athletes were more sensitive to provocation and significantly more likely to respond aggressively by stealing points from their opponents compared to noncontact athletes. Although the authors suggest that contact play causes aggression over time through positive reinforcement, it remains plausible that those electing to participate in high‐risk sports are more aggressive initially and participation reflects a personal preference for risky activities (Eysenck, Nias, & Cox, 1982). In fact, in Keeler's (2007) investigation, athletes who endorsed higher levels of aggression in their everyday lives were more likely to endorse hostile aggression in sports, while level of contact was not predictive of either form of aggression; thus, there may be bidirectional relationships between aggression and participation in sports.

It has also been shown that sports‐related activities serve as one of the leading causes of concussive injury, accounting for approximately 30% of all concussions sustained among youth (Langlois, Rutland‐Brown, & Wald, 2006). Not surprising perhaps, the incidence of concussion is higher in high‐risk sports (i.e., those that present a greater risk for injury such as hockey, football, or gymnastics) compared to low‐risk sports (e.g., basketball, baseball, and tennis; Beachy & Rauh, 2014; Gessel, Fields, Collins, Dick, & Comstock, 2007; Kerr et al., 2017; Marar, McIlvain, Fields, & Comstock, 2012; Noble & Hesdorffer, 2013). Indeed, in sporting leagues that permit contact play, the incidence of concussion is 3.88 times higher than that of noncontact leagues (Emery et al., 2010).

In any closed‐head injury, diffuse axonal damage results in a variable pattern of symptomatology; however, due to the increased vulnerability of the ventromedial prefrontal cortex (vmPFC)—a principal autonomic regulator—physiological underarousal at baseline is commonly observed (Fisher, Rushby, McDonald, Parks, & Piguet, 2015; Rushby et al., 2013, 2016). The vmPFC's location proximal to the orbital bones of the skull (Morales, Diaz‐Daza, Hlatky, & Hayman, 2007) renders it more susceptible to disruption following rapid acceleration/deceleration of the head; as a result, attenuated arousal is experienced across the spectrum of injury severity (Iverson & Lange, 2009)—even after milder injuries (Alcock, Gallant, & Good, 2018; Baker & Good, 2014; van Noordt & Good, 2011). Physiological underarousal, indexed by dampened respiration, electrodermal activation (EDA), and heart rate (Craig, 1968; Lazarus, Speisman, & Mordkoff, 1963), has been proposed as a mechanism of impulsivity and risk‐taking behavior. In particular, Hebb (1955) first suggested that individuals are attracted to risky activities when arousal levels are low because of their stimulating nature; Eysenck and Eysenck (1985) later expanded on this idea, applying the concept of underarousal to extraversion and proposing that this personality tendency represents a means of compensating for a dampened state. More recently, it has been demonstrated that healthy individuals who endorse higher levels of impulsivity exhibit physiological underarousal at rest (Mathias & Stanford, 2003) and lower resting heart rate levels have been associated with riskier responses compared to higher baseline levels (Schmidt, Mussel, & Hewig, 2013). Furthermore, it has been shown that individuals with dampened arousal experience greater sympathetic activity in response to sensation‐seeking or risk‐taking compared to those who are less impulsive (Houston & Stanford, 2001; Mathias & Stanford, 2003); as high‐risk athletes are more likely to sustain a concussion and be underaroused at baseline, aggression may be one possible means of increasing arousal and the prevalence of concussions among athletes may account for the increased aggression observed in sports.

Others have proposed that attenuated arousal after severe traumatic brain injury (TBI) leads to aggression due to physiological unpreparedness and an inability to anticipate negative outcomes (Bechara, Tranel, Damasio, & Damasio, 1996). According to the somatic marker hypothesis (Damasio, 1994), injury to the vmPFC disrupts the regulation of somatic cues that signal danger and emotionally relevant information. For example, van Noordt and Good (2011) showed that university students with a history of concussion exhibit lower EDA amplitude in anticipation of risky gambling decisions compared to their noninjured counterparts. In the absence of these biasing physiological markers, these individuals are less impacted by negative outcomes and more likely to engage in risky behaviors, such as substance abuse (Verdejo‐Garcia, Pérez‐García, & Bechara, 2006). When unexpected events occur, therefore, this reduced capacity to anticipate renders individuals more vulnerable to a heightened startle response which may be reflected behaviorally as aggression.

To complicate matters, it has been proposed that high‐risk athletes are arousal seekers, continuously pursuing opportunities for stimulation and engagement (Jack & Ronan, 1998; Kerr, 1991; Potgieter & Bisschoff, 1990; Rowland, Franken, & Harrison, 1986; Zuckerman, 1983) which may account for the higher levels of aggression observed postinjury. Indeed, Thompson and Morris (1994) found that increased anger, decreased attention, and increased stressful life events were significant predictors of injury susceptibility in football players. In addition, Greve et al. (2001) examined premorbid and postinjury levels of aggression in 45 individuals with a history of severe TBI; fourteen of the 26 individuals who exhibited impulsive aggression at the time of testing had a preinjury history of aggressive behavior, implying that premorbid personality may be a significant predictor of postinjury aggression. However, the authors did not examine whether aggression significantly increased from preinjury levels nor whether postinjury severity impacted postinjury aggression.

The aim of this research was to investigate the relative influence of athletic status and concussion history on aggression in high‐risk, low‐risk, and nonathletes. In particular, we examined physiological underarousal as a potential mediator of reactive aggression. It was hypothesized that athletes would endorse more aggression compared to nonathletes and that individuals with a history of concussion would endorse higher levels of aggression compared to those without a prior concussion, independent of athletic status. Consistent with the continuum of injury severity (Iverson & Lange, 2009) and previous research (e.g., Baker & Good, 2014; van Noordt & Good, 2011), those with a history of concussion were expected to exhibit lower levels of baseline physiological arousal (i.e., attenuated EDA) compared to those without a prior concussion. Lastly, as per the somatic marker hypothesis (Damasio, 1994), it was predicted that physiological arousal would mediate the relationship between injury severity and aggression whereby those with more severe concussive impacts would exhibit lower levels of arousal and subsequently higher levels of reactive aggression.

## 2 METHODS

### 2.1 Participants

Seventy‐seven university students were recruited to participate in this research. Participants were not recruited on the basis of head injury status to avoid “diagnosis threat” (see Suhr & Gunstad, 2002, 2005); thus, participants did not enter the study with complaints of postconcussive symptoms and were not aware of the researchers’ particular interest in this factor. To capture possible differences in premorbid personality, participants were categorized into one of three categories (high‐risk, low‐risk, or nonathlete) based on the primary sport listed for current participation in university athletics (recreational or competitive). Specifically, in accordance with previous investigations (Gallant, Barry, & Good, 2018), high‐risk sports (e.g., Noble & Hesdorffer, 2013) were defined as those that pose a greater risk of sustaining an injury due to the inherent nature of the sport. High‐risk sports included ice hockey, rugby, soccer, wrestling, lacrosse, gymnastics, powerlifting, martial arts/karate, and cheerleading, while low‐risk sports included basketball, volleyball, rowing/kayaking, swimming, track and field, baseball, curling, ultimate frisbee, fencing, and flag football.

Participants with a reported psychiatric condition were excluded from the analyses (*n *=* *11; *n *=* *2 high‐risk athletes; *n *=* *3 low‐risk athletes; *n *=* *6 nonathletes) given that psychiatric illness has a significant impact on personality, aggression (e.g., Starcevic, Uhlenhuth, Fallon, & Pathak, 1996), and levels of physiological arousal (Dienstbier, 1989; Fisher, Granger, & Newman, 2010). Thus, the analyses were conducted on 66 individuals (42.4% male; *M*_age_ = 20.91 years, *SD* = 2.64). Based on the criteria outlined in Gallant et al. (2018), 18 participants self‐identified as nonathletes, 24 as low‐risk athletes, and 24 as high‐risk athletes. Twenty‐seven participants (41% of sample) reported a history of concussion—the majority of which were athletes (see Table 1)—and the average time since injury was 2.75 years (*M*_months_ = 32.96). In addition, it was found that the majority of injuries were sustained during sports‐related activities (*n *=* *22; 81.5%), with hockey (36.4%) and rugby (13.6%) accounting for the largest proportion of sports concussions.

**Table 1:** Frequencies by athletic status and concussion history (*N *=* *66)

Athletic status	No concussion		Concussion		Total	
	*n*	%	*n*	%	*n*	%
Nonathlete	14	21.2	4	6.1	18	27.3
Athlete	25	37.9	23	34.8	48	72.7
Low‐risk	19	41.4	5	7.6	24	36.4
High‐risk	6	9.1	18	27.3	24	36.4
Total	39	59.1	27	40.9		

### 2.2 Materials

#### 2.2.1 Physiological arousal

Due to its increased sensitivity (Critchley, 2005; Fowles & Schneider, 1974), EDA was used as an index of changes in sympathetic nervous system activation—namely eccrine sweat gland activity. EDA amplitude was measured in microsiemens (μS) using Polygraph Professional Suite Software (Limestone Technologies, 2008). The Datapac USB 16‐bit Data Acquisition Instrument and a 16″Acer Laptop computer were used for collection. Silver‐silver chloride plated pads were placed on the distal phalynx of the index and fourth fingers of participants’ nondominant hands. Baseline was considered to be the average tonic level of an individual's response while in the absence of any discrete environmental event or external stimulus. All EDA data were manually filtered to exclude artifacts due to movement or physiological body actions (e.g., coughing, sneezing) using Polygraph Professional Suite Software.

#### 2.2.2 The wide range achievement test‐IV (WRAT‐IV)

The Word Reading and Spelling subtests of the WRAT‐IV Blue Form (Wilkinson & Robertson, 2006) were administered to obtain a proxy of intellectual capacity. In particular, the WRAT‐IV was administered to ensure that individuals were equally cognitively competent and to rule out any potential complications due to mild cognitive impairment. The Word Reading subtest assesses word recognition and knowledge by asking participants to read aloud a list of words. The Spelling subtest measures the capacity to translate sounds into written form and one's general word knowledge. Accuracy scores were calculated for both subtests.

#### 2.2.3 The everyday living demographic questionnaire (ELQ)

The ELQ (Good, 2015) was administered to collect a variety of information, including age, sex, level of education, lifestyle (e.g., sleep habits, recreational drug use), and other health‐related information. Notably, embedded within this questionnaire, participants are asked about a history of concussion as per the American Congress of Rehabilitation Medicine (ACRM) criteria (Kay et al., 1993; i.e., “have you ever sustained an injury to the head that was sufficient to produce an altered state of consciousness [ASC]? e.g., feeling dazed, dizzy or confused”). This definition is consistent with that of the Concussion in Sport Group (CISG) consensus statement (McCrory et al., 2017) which defines a concussion as a “direct blow to the head, face, neck, or elsewhere on the body with an impulsive force transmitted to the head … [resulting] in a range of clinical signs and symptoms that may or may not involve loss of consciousness.” Importantly, participants were also asked to provide a written description of the impact‐causing event to ensure that head injuries reported on the ELQ were those acquired via a traumatic, closed‐head impact.

Those who endorsed a history of concussion were asked to provide further information on the nature of the injury and a composite variable for injury severity was created by adding the severity indicators listed in Table 2. In particular, the following self‐reported endorsements were summed to form the composite: history of concussion (no = 0, yes = 1), transient loss of consciousness (TLOC—Owings et al., 1998; Rogers & O'Flynn, 2011; no = 0, yes = 1), duration of TLOC (<5 min = 1, <30 min = 2, <24 hr = 3), diagnosed concussion (no = 0, yes = 1), required stitches (no = 0, yes = 1), medical treatment received (no = 0, yes = 1), injury resulted in overnight stay at a medical facility (no = 0, yes = 1), symptoms lasting more than 20 min (no = 0, yes = 1), and multiple concussive injuries (no = 0, yes = 1). The reporting of similar indicators has been demonstrated to be a valid indicator of injury severity (Iverson & Lange, 2009; Jennett, 1976; Malec et al., 2007), especially given that neuroimaging techniques often lack the sensitivity to diagnose concussion and/or indicate injury severity (Bigler, 1999, 2013; Bigler & Brooks, 2009; Bigler & Orrison, 2001). Further, information relating to alcohol consumption was also gathered by the ELQ given that alcohol use in athletes is correlated positively with verbal and physical aggression (Dietze, Fitzgerald, & Jenkinson, 2008; Nelson & Wechsler, 2001).

**Table 2:** Injury severity indicators of self‐reported concussions by athletic status (*n *=* *27)

Injury severity indicators	High‐risk athletes (*n* = 18)		Low‐risk athletes (*n* = 5)		Nonathletes (*n* = 4)	
	*n*	% of total	*n*	% of total	*n*	% of total
Loss of consciousness (TLOC)	7	25.9	1	3.7	3	11.1
Duration of TLOC						
<5 min	4	14.8	0	0	1	3.7
<30 min	2	7.4	0	0	0	0
<24 hr	1	3.7	0	0	0	0
Diagnosed concussion	15	55.6	4	14.8	3	11.1
Required stitches	1	3.7	1	3.7	0	0
Received medical treatment	12	44.4	3	11.1	1	3.7
Overnight at medical facility	2	7.4	1	3.7	0	0
Symptoms for 20+ min	16	59.3	4	14.8	3	11.1
More than one injury	5	18.5	2	7.4	3	11.1

#### 2.2.4 Buss & Perry Aggression Questionnaire (BPAQ)

The BPAQ (Buss & Perry, 1992) is a 29‐item questionnaire that assesses self‐reported aggression using a five‐point Likert scale (from “extremely uncharacteristic of me” to “extremely characteristic of me”). The BPAQ consists of four subscales (Hostility, Anger, Verbal Aggression, and Physical Aggression); the Physical Aggression subscale is of particular interest in this study as an index of reactive aggression. As mentioned, reactive aggression refers to behaviors which are engaged in impulsively in response to perceived provocation or threat (e.g., Stanford, Houston, Mathias, Villemarette‐Pittman, Helfritz, & Conklin, 2003); as such, reactive aggression is not planned or intentional and can be captured by the Physical Aggression subscale. Specifically, the BPAQ physical aggression subscale includes items such as, “Once in a while I can't control the urge to strike another person” and “Given enough provocation, I may hit another person” which capture emotionally driven aggressive responses. Further, the association between impulsivity and physical aggression has been well documented (e.g., Standford et al., 2003; Hatfield & Dula, 2014), further supporting this conceptualization.

### 2.3 Procedure

This research received clearance from the Brock University Bioscience Research Ethics Board (#13‐310) and was conducted at the Lifespan Development Research Centre. Prior to participation, informed consent was provided. Each participant first completed one baseline recording of EDA. The signal was calibrated before each session to detect activity in the range of 0 to 20 μS; sampling started after a brief adjustment period during which the participant was asked to sit calmly and assume a comfortable position seated across from the examiner. Recording was terminated at the end of the 3‐min duration. During this recording, participants were instructed to sit as quietly and as still as possible with their eyes open. Finger electrodes were applied, and EDA was recorded using the traditional continuous voltage method (see Lykken & Venables, 1971). After electrode removal, all participants completed the WRAT‐IV, followed by the self‐report questionnaires described above. Participants were then debriefed and thanked for their time.

### 2.4 Data analyses

Analyses were conducted using the Statistical Package for the Social Sciences, version 22 (SPSS, http://scicrunch.org/reslover/SCR_002865). All assumptions have been examined and met unless otherwise stated. Statistical analyses were conducted without sex as a covariate, as the inclusion of this factor did not significantly affect results. Further, although Buss and Perry (1992) report that men score significantly higher than women on the Physical Aggression scale of the BPAQ, preliminary analyses revealed no sex differences across all but one (Verbal Aggression) of the subscales (see Table 4). Pearson Chi‐square tests were used to examine associations among athletic status (nonathlete, high‐risk athlete, low‐risk athlete) and concussion history (concussion, no‐concussion), while hierarchical multiple linear regressions were used to predict aggression and physiological arousal from a number of predictor variables, such as concussion history (concussion, no‐concussion) and athletic status (high‐risk, low‐risk, and nonathletes; athlete, nonathlete). Multiple regressions were conducted to analyze the proposed mediation model, as per the Baron and Kenny (1986) approach.

## 3 RESULTS

### 3.1 Demographic information

There were no significant differences in intellectual capacity between those with and without a history of concussion as observed on the Spelling subtest (total score), *F*(1, 59) = 2.07, *p *=* *0.280, and the Word Reading subtest (total score), *F*(1, 60) = 3.87, *p *=* *0.186, of the WRAT‐IV. In addition, no significant differences in Spelling (*p *=* *0.209) or Word Reading (*p *=* *0.938) performance were observed as a function of athletic status (nor when it was dichotomized as athlete, nonathlete; *p *>* *0.05).

A 2 × 3 Chi‐square test of independence was performed to examine the relation between athletic status and a history of concussion. The relation between these variables was significant, *χ* ^2^(2) = 18.14, *p *<* *0.001, such that high‐risk athletes were more likely to have a history of concussion than were low‐risk and nonathletes. Further, a one‐way ANOVA showed that the number of previously sustained concussions differed among athletic groups, *F*(2, 63) = 4.16, *p *=* *0.020. Post hoc analyses using the Tukey HSD criterion for significance indicated that the average number of prior concussions was significantly higher in the high‐risk athlete group (*M *=* *1.71, *SD* = 2.42) compared to the low‐risk athlete group (*M *=* *0.33, *SD* = 0.76; see Table 1).

There were no major differences in health, psychosocial, and demographic information based on concussion history or athletic status (*p*'s > 0.05). Information relating to substance use was also examined as a function of athletic status and concussion history. There was no main effect of athletic status, *F*(2, 59) = 5.88, *p *=* *0.145; however, there was a main effect of concussion history, *F*(1, 59) = 28.92, *p *=* *0.014, in terms of the number of alcoholic drinks consumed per outing, such that those with a history of concussion drank significantly more.

### 3.2 Aggression as a function of athletic status and concussion history

Three participants were missing data (for alcohol‐related variables, *n *=* *2, or the BPAQ, *n *=* *1) for the following analyses. Pearson correlations revealed that the number of alcoholic drinks consumed per outing was related positively to injury severity, *r*(65) = 0.248, *p *=* *0.046, and the Physical Aggression subscale of the BPAQ, *r*(64) = 0.354, *p *=* *0.004; as such, alcohol consumption per outing was entered in the first stage of all subsequent hierarchical multiple regression analyses. Thus, a series of three‐step hierarchical multiple regressions were conducted with total scores on the BPAQ and each of its subscales as dependent variables with athletic status (athlete, nonathlete) entered in step two, and concussion history (concussion, no‐concussion) entered in step three.

Across all aggression variables, athletic status was not a significant predictor, *p > *0.05. However, concussion history, and injury severity in general, significantly predicted Physical Aggression over and above alcohol consumption and athletic status, whereby those with a history of concussion reported significantly higher levels of physical aggression (see Table 3). No further analyses were conducted examining aggression as a function of athletic status. Means and standard deviations for all subscales of the BPAQ are included in Table 4 as a function of athletic status.

**Table 3:** Hierarchical regression analyses for concussion status and injury severity predicting physical aggression (*N *=* *63)

Variables	Model 1			Model 2			Model 3			Model 3		
	*B*	*SE B*	*β*	*B*	*SE B*	*β*	*B*	*SE B*	*β*	*B*	*SE B*	*β*
Drinks per outing	2.32	0.779	0.354**	2.19	0.794	0.334**	1.84	0.796	0.280*	1.78	0.797	0.271*
Athletic status				1.86	2.17	0.104	1.12	2.15	0.063	1.26	2.12	0.070
Concussion status							3.92	1.96	0.244*	—	—	—
Injury severity										0.755	0.340	0.267*
*R* ^2^		0.125			0.135			0.189			0.201	
*F* for change in *R* ^2^		8.86**			0.732			3.99*			4.92*	

*Notes*

DV: Physical aggression (Buss & Perry Aggression Questionnaire Subscale; Buss & Perry, 1992).

*p <* *0.05, **p <* *0.01.

**Table 4:** Means and standard deviations for the Buss & Perry Aggression Questionnaire (BPAQ) by athletic status and sex (*N *=* *66)

	Anger	Hostility	Verbal aggression	Physical aggression	Total
	*M (SD)*				
Nonathlete	17.2 (4.4)	21.8 (5.4)	14.6 (3.6)	18.4 (6.2)	71.9 (15.9)
Athlete	15.6 (5.9)	20.3 (5.5)	15.0 (4.2)	21.2 (8.4)	71.4 (20.1)
Low‐risk	14.9 (5.4)	20.0 (5.7)	14.0 (3.9)	19.4 (7.9)	66.6 (18.8)
High‐risk	16.3 (6.4)	20.6 (5.4)	15.9 (4.4)	23.1 (8.6)	76.2 (20.6)
Male	15.4 (5.7)	20.7 (5.7)	16.0 (3.7)	21.9 (8.8)	72.1 (19.6)
Female	16.5 (5.5)	20.7 (5.3)	14.0 (4.1)	19.3 (7.1)	71.1 (18.6)

### 3.3 Concussion history and physiological arousal

Hierarchical multiple regressions were conducted with athletic status entered in the first step and concussion history entered in the second step to predict baseline EDA amplitude. These analyses revealed that concussion history significantly predicted EDA amplitude independent of athletic status, whereby those with a previous head injury exhibited significantly lower levels of baseline physiological arousal compared to those without a prior concussion, *B *=* *−0.946, *SE B *=* *0.429, *β* *= *−0.276, *p *=* *0.031. Athletic status did not significantly predict EDA, *β* *= *0.002, *p *=* *0.985. Similarly, further analyses using injury severity on step two showed a trending effect and uniquely accounting for 5.4% of the variance in EDA, *B *=* *−1.81, *SE B *=* *−0.088, *β* *= *−0.237, *p *=* *0.063.

### 3.4 Injury severity, physiological arousal and aggression

**Figure 1:**
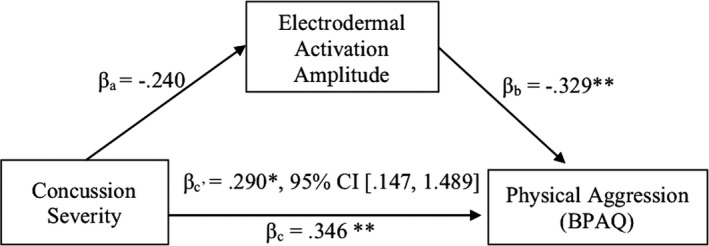
Indirect effects of electrodermal activation amplitude on the relationship between concussion severity and physical aggression on the Buss Perry Aggression Questionnaire (BPAQ), *p < 0.05, **p < 0.01

To investigate underarousal as a mechanism of physical aggression, a meditation analysis was conducted. Given that alcohol consumption was not associated with the proposed mediator, it was not included as a covariate in the mediation analyses. As Figure 1 illustrates, the standardized regression coefficient between injury severity and EDA amplitude was marginally significant (*p *=* *0.054) and the standardized regression coefficient between physiological arousal and physical aggression was statistically significant (*p *=* *0.008), as was the indirect effect of injury severity on physical aggression (*β* *= *0.290, *p *=* *0.018). Noting that the unstandardized indirect effect was 0.818, 95% CI [0.147, 1.489], the indirect effect was statistically significant.

## 4 DISCUSSION

The current study examined athletic status and concussion history as predictors of aggression. Previous research has shown that athletes exhibit higher levels of sensation‐seeking, aggression, and competitiveness (Ahmadi, Besharat, Azizi, & Larijani, 2011; Potgieter & Bisschoff, 1990; Zuckerman, 1983) and therefore may be more susceptible to injury (e.g., Kerr et al., 2017; Thompson & Morris, 1994). As expected, high‐risk athletic status was associated with a history of concussion: three‐quarters of those identifying as high‐risk athletes reported at least one previous concussion. While it remains unknown whether high‐risk athletes were physiologically underaroused and aggressive prior to their concussive injuries, the current findings challenge this interpretation. Although researchers have often proposed that the instrumental value of aggression in sports serves to reinforce aggressive tendencies (Butt, 1987; Weiner, 1974), athletic status was not found to be a significant predictor of self‐reported aggression in the current study. This finding is in line with the work of Lemieux, McKelvie, and Stout (2002) which revealed no differences in aggression between athletes and nonathletes when they were matched on physical size.

Concussion history was a significant predictor of self‐reported aggression; those with a prior concussion endorsed higher levels of physical aggression, as opposed to overall aggression, verbal aggression, hostility, or anger. Importantly, when investigating potential mechanisms of this relationship, it was found that physiological arousal was a partial mediator of the relationship between injury severity and physical aggression, such that those with more severe injuries exhibited lower levels of physiological arousal, which in turn accounted for higher levels of aggression. Consistent with previous work indicating physiological dysregulation after concussion (e.g., Alcock et al., 2018; Baker & Good, 2014; van Noordt & Good, 2011), these findings imply that aggressive behaviors may be associated with concussion history (i.e., physiological underarousal), over and above athletic status, due to physiological unpreparedness. Alternatively, due to their chronically underaroused state, these athletes may be more likely to engage in aggressive forms of play in an attempt to increase their baseline levels of alertness; however, the current study did not directly test these potential explanations.

Although reactive aggression has previously been associated with a heightened physiological state (Zillmann, 1971), the somatic marker hypothesis would emphasize that it is also associated with a lowered physiological anticipatory state (e.g., Bechara et al., 1996). Indeed, Bechara et al. point out that emotional dysregulation is a hallmark of TBI due to the vmPFC's reduced capacity to regulate and elicit anticipatory somatic signals. Consistent with this view, in the current study participants who exhibited lower levels of arousal endorsed more items relating to overreaction (e.g., “Once in a while, I can't control the urge to strike another person”) compared to items of proactive aggression (e.g., “I often find myself disagreeing with people”).

### 4.1 Limitations

It is important to acknowledge certain limitations of the study. First, concussion history was determined via retrospective self‐report; thus, many of the reported injuries remain unconfirmed by collateral sources. However, it has been found that the majority of university students reporting concussions indicate that they were not admitted to a treating medical facility for their injuries (e.g., Baker & Good, 2014; Segalowitz & Lawson, 1995), thereby limiting collateral evidence, and the possible impact of retrospective reporting bias is somewhat mitigated in this study in that participants were not motivated by litigation or incentives, nor were they aware of the researchers’ added interest in head injury upon recruitment. Further, while the majority of injuries captured in this study were mild in nature according to injury severity indices, it is acknowledged that the ACRM criteria allow for moderate and severe TBI classifications to fall into this group; thus, this research may include a broader spectrum of individuals across the continuum and may not be restricted to the mild category. However, moderate severity includes LOC greater than 30 min in duration (Blyth & Bazarian, 2010) and only one student in the current sample met that criterion.

It should be noted that the majority of participants in this sample are female (60%) and within‐sport comparisons have shown that self‐reported injuries are higher in females (for a summary, see Graham, Rivara, Ford, & Spicer, 2014). Moreover, the authors acknowledge that some of the sample groups were very small (i.e., the high‐risk, no‐concussion group) and that larger sample sizes would have provided greater power for statistical analyses.

### 4.2 Implications and future directions

The findings from this study demonstrate that concussion history and severity are negatively associated with physiological arousal and provide preliminary evidence of a link between physiological arousal and physical aggression in university athletes. The authors hypothesize that this subtle, dampened arousal state may render those with a concussion history more vulnerable to aggressive overreaction, particularly under conditions of uncertainty; however, further research is needed to directly assess the aggressive reactions of individuals with a prior concussion in response to unanticipated provocation and to examine whether this behavior can be attributed to their lessened anticipation of unexpected events.

Previous research has shown that vmPFC damage is associated with emotional dysregulation and overreaction in the face of unanticipated events (Bechara et al., 1996); thus, dampened physiological feedback after concussion may be a mechanism of reactive, rather than proactive, aggression among athletes. Individuals with a history of concussive injury may be more likely to engage in reactive aggression given that it is defined as an act of retaliation and is performed in response to perceived threat or provocation (Anderson & Bushman, 2002). In particular, this physiological dysregulation may serve as an indicator of a reduced capacity to monitor and therefore predict, one's environment. According to Brown and Ryan (2003), individuals naturally exhibit differences in the propensity to be engaged with, and responsive to, their surroundings; this personal disposition is referred to as mindfulness (Brown, Goodman, & Inzlicht, 2012), and it has been associated with increased sympathetic arousal (Lumma, Kok, & Singer, 2015), as well as greater self‐regulation (Short, Mazmanian, Oinonen, & Mushquash, 2016). Notably, Krzeczkowski, Robb, and Good (2017) found that individuals with a history of concussion who endorsed greater trait mindfulness presented with fewer postconcussive symptoms. Thus, individuals with lower baseline arousal may be more vulnerable to overreaction to any unexpected experience due to a reduced capacity to monitor their environment and therefore effectively regulate their behavioral responsivity, relative to their cohort.

These findings implicate concussion as an important predictor of self‐reported aggressive behavior. In addition, the current study mirrors findings of underarousal and reactivity in the TBI literature and implies that a history of concussion is also related to affective regulation. As the current sample consisted of asymptomatic university students, these findings suggest that the effects of subtle disruption after concussion are associated with changes in physiological functioning and behavior. Overall, the assessment of self‐reported concussion history may be an important inclusion criterion in future investigations examining aggressive tendencies in sports. Further research is needed to elucidate the precise nature of this relationship and to examine changes in underarousal and reactive aggression using longitudinal methods.
